# Physical activity calorie equivalent (PACE) food labelling on discretionary foods in secondary school canteens in England: an efficacy cluster randomised controlled trial

**DOI:** 10.1186/s12966-025-01710-1

**Published:** 2025-02-26

**Authors:** Natalia Iris, Fehmidah Munir, Amanda J. Daley

**Affiliations:** 1 Centre for Lifestyle Medicine and Behaviour, School of Sport, Exercise and Health Sciences, Loughborough University, Loughborough, LE11 3TU UK; 2 School of Sport, Exercise and Health Sciences, Loughborough University, Loughborough, LE11 3TU UK

**Keywords:** Food labelling, PACE labelling, Calories, Food choice, Adolescents, Children, Schools, School canteens, Discretionary food

## Abstract

**Background:**

Schools do not typically implement food labelling in their canteens, therefore young people may not be given nutrition information on which to make their food choices. One way of expressing the energy/calorie content of foods is to provide this information in the form of physical activity calorie equivalent (PACE) food labelling, which may help to contextualise the energy content of food/drinks to young people in a simple and understandable way. The study aimed to assess the usefulness of implementing PACE labelling in school canteens and to conduct a process evaluation of using this type of food labelling with young people.

**Methods:**

A parallel two-armed cluster RCT to evaluate a PACE labelling intervention in secondary schools (typically, adolescents aged 11 and above) in England was conducted. Schools were randomised on a 2:1 basis to display PACE labelling by cakes/sweet biscuits in canteens or to continue with usual practice (comparator) for up to six weeks. There was a baseline period of no PACE labelling for a minimum of four weeks in all schools. Anonymised purchase data were provided by schools and analysed both descriptively and using analysis of covariance.

**Results:**

Eighteen schools in England were randomised and 11 participated (6 intervention and 5 comparators). Analyses are based on ~ 99,000 purchase transactions of cakes and biscuits from participating schools. There was a reduction in cake/biscuit purchases in intervention schools versus comparators of ~ 11 items per week per 100 students at follow-up (adjusted mean difference = -0.112, 95% CI [-0.179 to -0.045], *p* = 0.005). Intervention schools did not report major difficulties with the implementation of PACE labelling.

**Conclusions:**

PACE labelling appeared to reduce cakes/biscuit purchases by a small amount and may be a useful approach to reducing the purchase of discretionary foods in young people in the school environment. The implementation of PACE labelling appeared feasible for some schools, but other schools had reservations about the adverse effects this type of labelling may have on the well-being of students.

**Trial registration:**

Registered on ClinicalTrials.gov on 18th November 2022. NCT05623618, https://clinicaltrials.gov/study/NCT05623618.

**Supplementary Information:**

The online version contains supplementary material available at 10.1186/s12966-025-01710-1.

## Background

Childhood obesity continues to be a serious health concern which can lead to poor health outcomes throughout life [1]. Worldwide, 20% of children and adolescents (aged 5 to 19 years) were overweight or obese in 2022 [1]. In England, it is estimated that around 40% of children are living with overweight or obesity by the time they leave primary school [2]. Living with excess weight increases the risk of long-term conditions [1], and obesity in childhood can continue into adulthood [3].

Evidence suggests young people are consuming an excessive number of discretionary and ultra-processed foods such as cakes, biscuits, confectionary and sugary drinks [4, 5]. Discretionary food/drinks are energy dense, provide little nutritional value and can contribute to poor health outcomes [6]. Discretionary foods such as cakes and biscuits are widely available for young people to consume and in many countries, including England, these types of foods are permitted to be sold in secondary schools [7].

In England, food labelling is displayed on packaged food/drinks using traffic light labelling [8] and non-packaged food/drinks in out of home settings (e.g. in restaurants and cafes) using absolute calorie labelling [9]. Calorie labelling is considered a way to promote healthier food/drink choices and it is implemented in several countries in out of home settings [10]. However, in many countries schools do not implement labelling in food environments, including school canteens, therefore young people may not be given nutrition information on which to make their food choices every day at school.

A recent review has indicated that the effects of calorie labelling on food selections are small [11]. Nutrition labelling may not influence food/drink choices in young people because it is difficult to understand, which may be due to not developing complex thinking processes until late adolescence [12]. This highlights that food labelling approaches that contain more accessible information are urgently required to help guide young people with their food choice decisions.

**Fig. 1:**
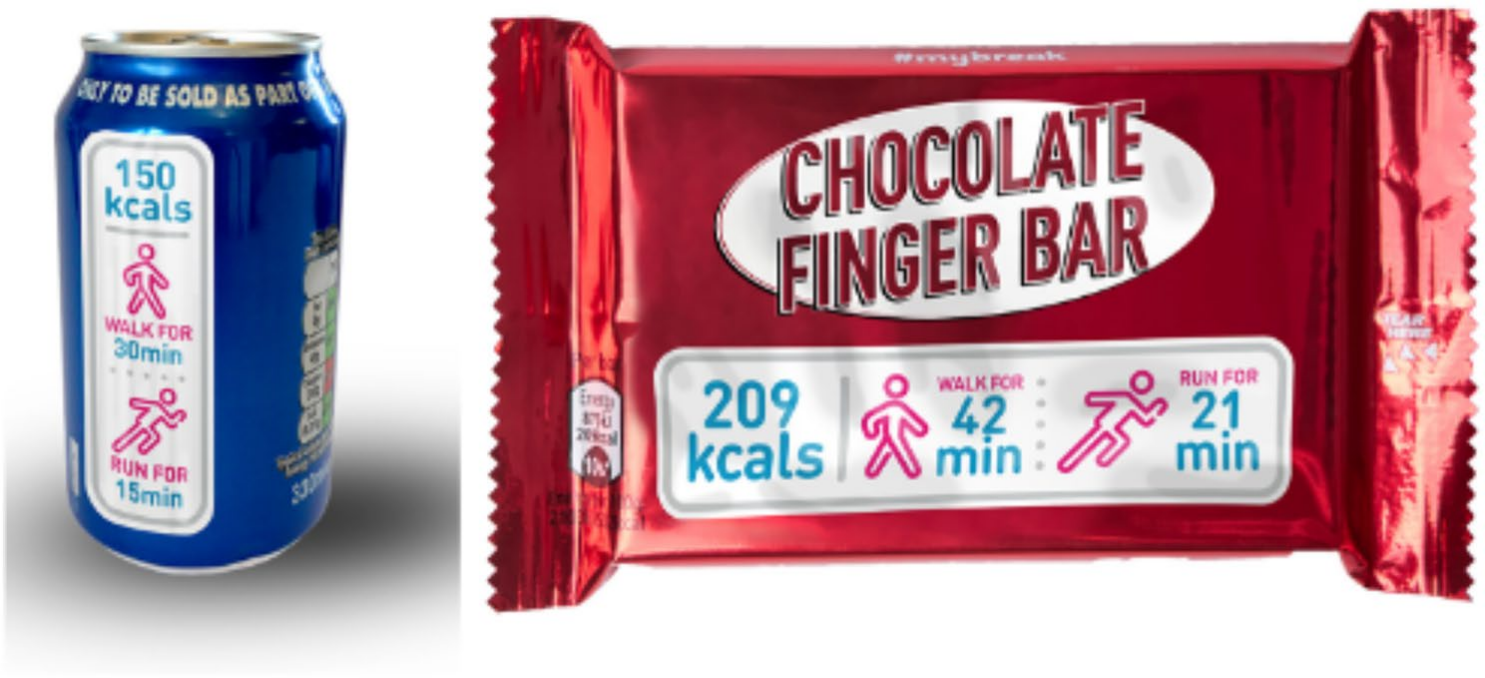
Examples of PACE labelling [29]

Physical activity calorie equivalent (PACE) food labelling aims to contextualise the energy/calorie content of food and drinks by displaying the number of minutes or miles/kilometres of physical activity equivalent to the calories contained in a food/drink item. For example, a muffin of 600 calories may take an adolescent weighing 52 kg around an hour of running to expend the calories it contains (see Fig. 1 for examples) [13]. This type of labelling may nudge young people in their decision making when choosing food/drinks, while also being a means of encouraging physical activity. There is some evidence to suggest that PACE labelling may influence food/drink choice [14, 15], including among adolescents [16]. Most studies to date have tested PACE food labels in laboratory settings and/or hypothetical food choice scenarios in adults [14]. There is limited research with young people, but a recent study has indicated that adolescents in the United Kingdom (UK) may find PACE labelling easier to understand and more useful than traffic light labels [17]. Qualitative evidence in the United States of America has also reported that adolescents may prefer PACE labelling over other food labelling approaches because it displays information in a more meaningful way to them [18].

Given the evidence that PACE labelling may impact food selection/consumption [14–16] and could be appealing to young people [17, 18], it is now important to test the merits of this approach to food labelling in real life settings, where discretionary foods are routinely available to young people. School canteens in secondary schools are used by young people every day where discretionary foods are purchased making this an important context in which to evaluate different approaches to food labelling. The present study aimed to evaluate the usefulness of PACE labelling on reducing discretionary food purchases (cakes and sweet biscuits) in secondary school children. Specifically, this study aimed to assess whether PACE labelling points to reducing the selection of discretionary food purchases in young people prior to conducting a definitive trial. The findings could help guide future health policy on PACE labelling as a population health strategy in young people in school settings and other contexts.

## Methods

### Study design

**Fig. 2:**
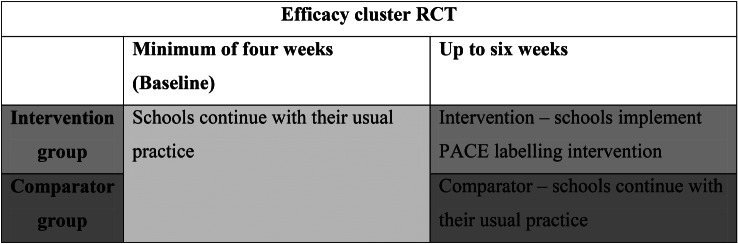
Overview of study design

A parallel two-armed cluster randomised controlled trial (RCT) that tested a PACE labelling intervention in secondary schools was conducted. Schools were randomised to display PACE labelling (for up to six weeks) on at least one cake/sweet biscuit item, or continue with usual practice (comparator), within canteens. There was a baseline period of no PACE labelling for a minimum of four weeks in all schools. See Fig. 2 for the study design. Anonymised purchase data on the number of cakes/sweet biscuits sold in the study periods were collected from schools.

To complement the trial, a nested process evaluation was conducted which included assessment of intervention fidelity and school stakeholder acceptability. Information was gathered through a fidelity present/absent assessment, feedback from school staff and project records/monitoring. A nested qualitative study with students in the intervention schools to explore their views of the PACE labelling was also conducted and this will be reported elsewhere. Favourable ethical approval for this study was obtained from Loughborough University’s Ethics Approvals (Human Participants) Sub-Committee (reference number: 7011). The trial was registered at ClinicalTrials.gov (ID: NCT05623618). The CONSORT checklist [19] and extension to cluster randomised trials [20] was used as a guide to report the study.

### Recruitment and participants

School trusts and secondary schools were contacted to invite them to participate in this research. The study was also promoted to schools through contacts/networks and via social media. Schools interested in taking part were sent a school study information sheet. This included a statement regarding the possibility that some parents may raise concerns about sensitive issues related to eating and physical activity in children.

There are varying catering/canteen set up options in schools in England. Generally, school meals tend to be provided either by internal caterers or by external catering companies. Food is usually available in a canteen where there is a queueing system (e.g. food is displayed, children queue and select/collect what they would like then purchase the meal/items from a cashier/till). In England, there are government approved School Food Standards that stipulate the types of foods that are permitted to be sold in schools [7]. Some types of discretionary foods are permitted in schools (with restriction depending on the type). For example, serving cakes, biscuits, desserts and pastries is permitted at lunch times, but these items should not be available at other times in the school day (e.g. morning break and in vending machines), unless they are yoghurt/fruit based desserts containing no less than 50% fruit [7]. Confectionary, chocolate and sugary drinks should not be served at any time across the school day [7].

The types of cakes, biscuits, desserts and pastries (and other permitted discretionary foods) offered varies by school and by day, and this depends on factors such as stock deliveries, ingredient availability and the space available in display areas in each school canteen. Based on the schools in this study, there can be at least four types of cakes/biscuits (which were the discretionary foods of interest) offered per day (and these can be offered in different flavours). For example, muffins, cookies, sponge cakes and flapjacks.

Interested schools were assessed for eligibility and information was collected on the cakes/sweet biscuits that were sold by them, the layout of the canteens/display areas and how purchases were recorded. The cakes/biscuits to be labelled in the study and how the PACE labelling would be displayed if the school was randomised to the intervention was agreed with each school. Each school was offered a £50 gift card to thank them for participating in the study, and administration costs (if any).

### School eligibility

Secondary schools (adolescents aged 10–19 years) wishing to participate in the study were included if they sold cakes and/or sweet biscuits for at least two days per week and sold the same cake(s)/sweet biscuit(s) before the study. Schools had to be able to provide anonymised purchase data for the selected study food items, specifically, the number and dates that selected study food items were sold. For a school to take part a PACE label needed to be practically placed near to the selected study cake(s)/sweet biscuit(s) in a school canteen used by students. Both headteachers (or another appropriate authority) and catering managers needed to consent for the school to take part. Schools were eligible to participate if they were not involved in any other school trials or initiatives related to food.

### Consent (parents and students)

Consent/assent from parents and students were not required as no personal or individual level data was collected (excluding the qualitative study, which will be reported elsewhere). Once the school consent procedures had been completed each school was asked to send the parents of their students information about the study. The parental information document stated that PACE labelling would be implemented in some schools and that if parents had any concerns about this to contact the lead researcher.

### Randomisation

Schools were the unit of randomisation (clusters). After schools had provided written consent to participate they were randomised to either the intervention (display PACE labelling) or the usual practice comparator group. An approach of 2:1 randomisation was used (intervention: comparator) as the focus of the study was on understanding the usefulness of PACE labelling on reducing purchases of cakes and biscuits and on the experiences of introducing PACE labelling within schools. Schools were randomised in batches of three after sufficient schools had consented. Randomisation was conducted by a researcher who had no other involvement in the study. Due to the nature of the study, it was not possible for the research team to be blinded to group allocation. Groups allocation was also known when conducting the statistical analyses of the study data.

### The PACE labelling intervention

#### Development and design of the PACE labelling

**Fig. 3:**
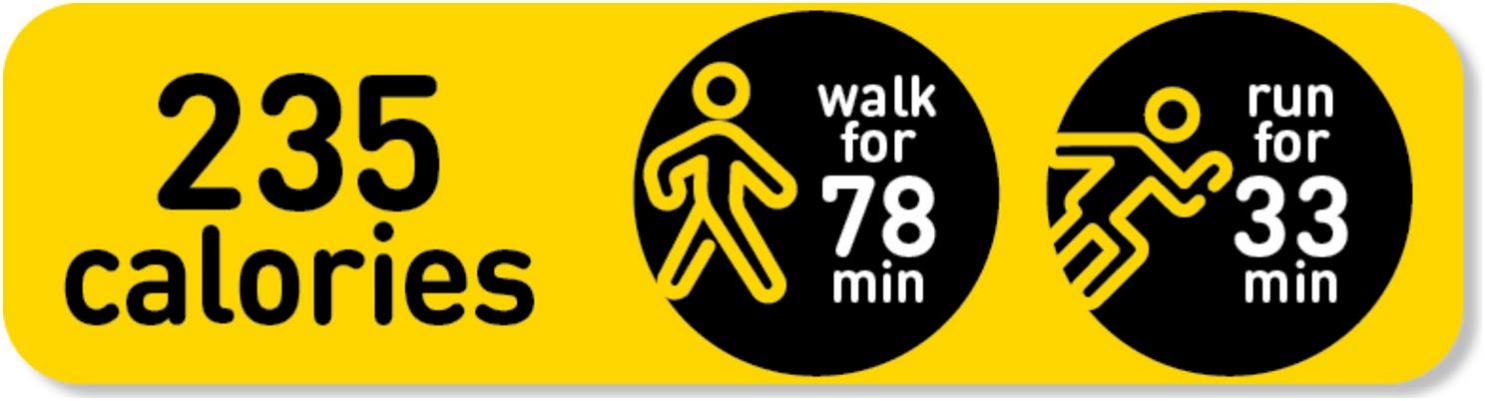
The PACE label design used in the intervention [From the authors/Loughborough University]

Detailed views about PACE labelling among young people was gathered in a prior mixed methods programme of research [17]. This research helped to create the PACE labelling intervention used in this trial so that it was developed using a theory and evidence-based approach. The intervention development was guided by several established frameworks including Intervention Mapping [21], the COM-B [22], and the Theoretical Domains Framework [23]. The PACE label design used in the study (see Fig. 3) displayed the number of calories of the food item with the equivalent number of minutes of walking and running to the calorie amount. All physical activity information on the labels was based on a 52 kg weight, which is the average weight of an adolescent aged 14.5 years [24].

In the schools allocated to the intervention, PACE labels were displayed near cakes/sweet biscuits sold to students in the school canteens for up to six weeks. The PACE labels were displayed near products either using plastic display boards or clips, or by labelling existing display stands/sneeze boards. The PACE labelling was visible to all students who used the canteen in the included schools.

#### Selection of discretionary foods to target in the intervention

The intervention targeted cakes and sweet biscuits as these are ultra-processed [25], discretionary and are permitted to be sold in secondary schools in England [7]. The aim was to target foods that were sufficiently similar to allow for comparison of items and consistency across/within schools, while recognising that schools vary in the cakes and sweet biscuits that are sold.

#### Intervention group procedures

In the intervention schools, calorie content information for the chosen cakes/biscuits were collected (or calculated) and the PACE information calculated using the World Cancer Research Fund online calculator [13]. The PACE food labelling was then produced for each school. School canteen staff were helped to set up the labelling and were asked to ensure the PACE label was displayed near the chosen cakes/biscuits every day during the intervention period. When the intervention period ended, the PACE labelling was removed.

### Comparator group

Schools allocated to the comparator condition were asked to continue with their usual practice.

### Purchase of cakes and biscuits (primary data of interest)

The main data of interest was the number of weekly (can be scaled to daily) purchases/units sold of selected cakes and biscuits at the school level. Purchasing was used as a proxy outcome for consumption given most individuals would expect to consume what they purchase, particularly for discretionary foods such as cakes and biscuits. Anonymised total daily/weekly purchase data for the number of cakes/sweet biscuits was collected from all schools. Purchase data recording was either electronic or manual. Purchase data were collected for the baseline period (a minimum of four weeks prior to the intervention period) and intervention period (up to six weeks) (or equivalent for comparators) in schools.

### Intervention fidelity

Fidelity checks were conducted for each intervention school to establish whether the PACE labelling intervention was delivered as intended (fidelity considered present/absent). Catering staff were regularly contacted to check the labelling was visible and in situ, and to resolve any problems. Fidelity checks consisted of observation visits, requests for photographs of the PACE labels in place and regular phone calls/messages with catering staff.

### Sample size

The sample size was determined by what was achievable within a fixed budget and within the limitations of what schools would be likely to commit to in terms of study duration. Together, this meant that the sample size for the study was fixed and therefore a power or sample size calculation was not conducted. Additionally, this study tested a public health intervention that would have a negligible cost to the public purse, where even small effects in the direction of benefit would be likely to be worthwhile. Consequently, sample size calculations based on realistic minimally clinically important differences would be unachievably large. Therefore, in studies such as these it is less important to articulate what might be considered a meaningful effect or target effect size.

### Data handling and analyses

Purchase data was excluded from analyses if it was considered that events on particular days would influence the number of purchases of cakes and biscuits (e.g. school closure days or snow days). If there were missing data dates, weekly data was estimated using extrapolation where possible (using pre-defined rules). As comparator schools did not have discrete baseline and intervention periods, the baseline (minimum of four weeks) and intervention periods were defined by dividing data to form these two discrete study periods. Whilst comparator schools did not have intervention, for ease and for comparisons this time period is referred to as the “intervention period”.

Data analyses were conducted using IBM SPSS version 28. Analyses were conducted at the school level. The percentage of pupils eligible for free school meals was used as a proxy for school level socio-economic position/deprivation and urban/rural description of schools was also collected [26]. The number of units of cakes/biscuits sold per day for baseline and intervention periods were calculated/collated for each school. Weekly purchase totals were calculated and then weekly averages of purchases were calculated from these data for the baseline and intervention period for each school. Absolute weekly purchase totals and average weekly purchase totals for each school were analysed. The above analysis was repeated including weekly average of purchases for the baseline and intervention period adjusted for school size (number of students). Average weekly rates of purchases were calculated per 100 students so that data could be expressed in a standardised way.

Analysis of covariance (ANCOVA) was conducted to assess whether there was a significant between group difference in the change in purchases between the two timepoints of baseline and intervention. The purchase rate during intervention phase was included as the dependent variable, the randomisation arm as the independent variable and baseline rate as a covariate. To allow for any extra variation due to cluster size, the regression was weighted by the number of pupils in each school. Sensitivity analyses were conducted which included or excluded purchase data from individual schools to assess impact on the between group difference.

## Results

**Fig. 4:**
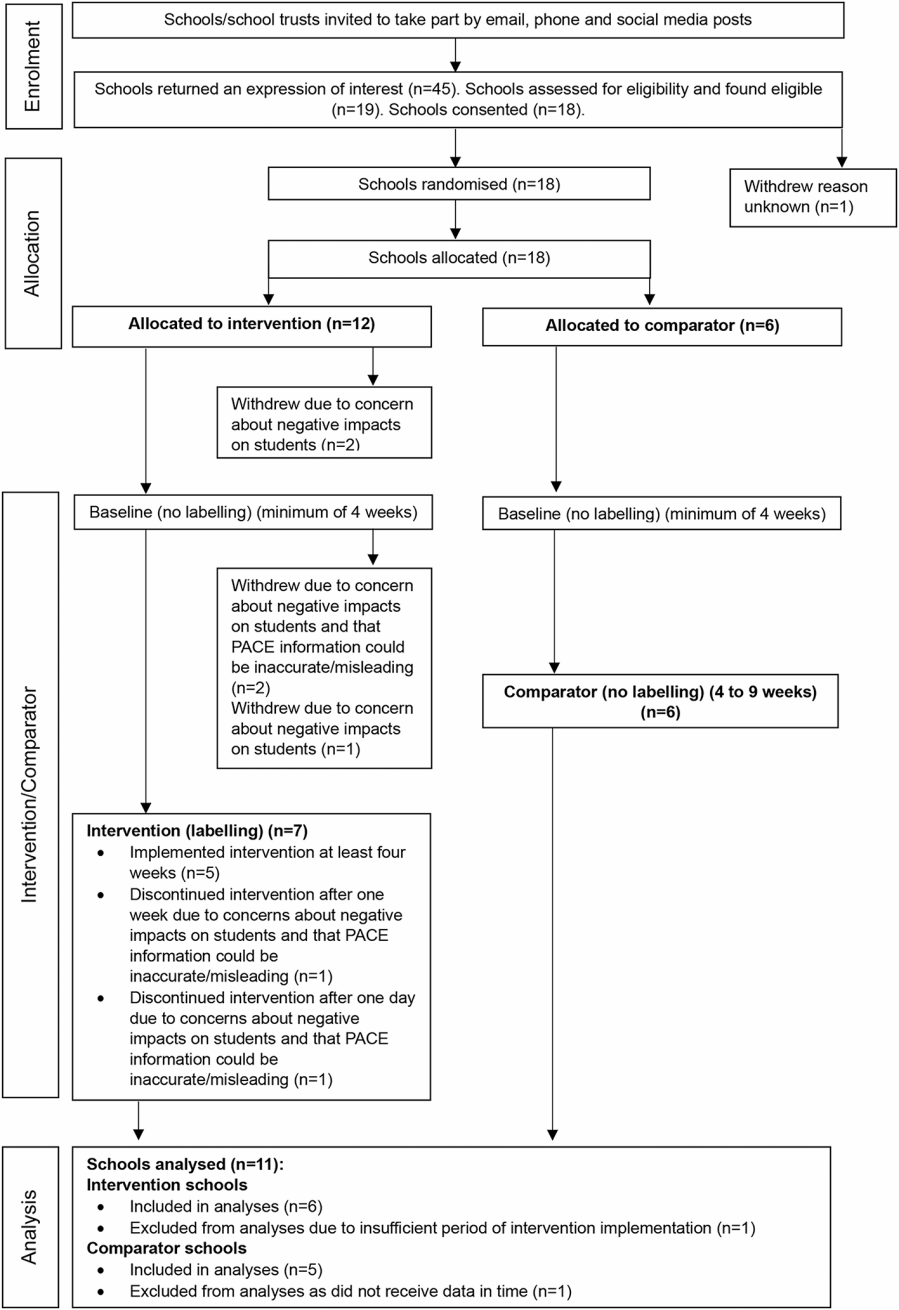
The flow of schools through the study

Figure 4 displays the flow of schools through the study. Recruitment of schools commenced from 28th February 2022. Data collection for baseline and intervention periods occurred between 5th September 2022 to 28th March 2023. Eighteen schools were randomised to the intervention (12 schools) or comparator group (six schools). Of the 12 schools that were randomised to the intervention group, five withdrew prior to implementing the labelling. The main reason given were due to concerns about negative impacts of the PACE labelling on the health and well-being of students.

The intervention was initiated up to three months after randomisation. Six of the seven intervention schools implemented the PACE labelling for at least one week and are included in the analyses (five schools implemented the intervention for at least five weeks and one school for one week). One school implemented the labelling for one day and has been excluded from the analyses. Of the six schools randomised to the comparator group, five provided purchase data and were included in the analyses.

### School characteristics

Schools ranged in size from ~ 700 to 1800 students, were located in urban (*n* = 8) and rural areas (*n* = 3), across the East and West Midlands and the North East regions of England. Schools were located in both affluent and deprived areas as defined by the percentage of pupils eligible for free school meals, which ranged from ~ 10–40%. The characteristics of schools, such as type of area/school, were similar in both trial groups.

### Descriptive purchase data collected in intervention and comparator schools

Data for 98,973 transactions (intervention schools = 49,041, comparator schools = 49,932) over the study period were included in the analyses. In intervention schools, the total number of purchases at baseline was 32,291 and the average school (cluster) size was 1,230 students. In comparator schools, the total number of purchases at baseline was 24,776 and the average school (cluster) size was 1,066.

### Purchases of cakes and biscuits in intervention and comparator schools

**Fig. 5:**
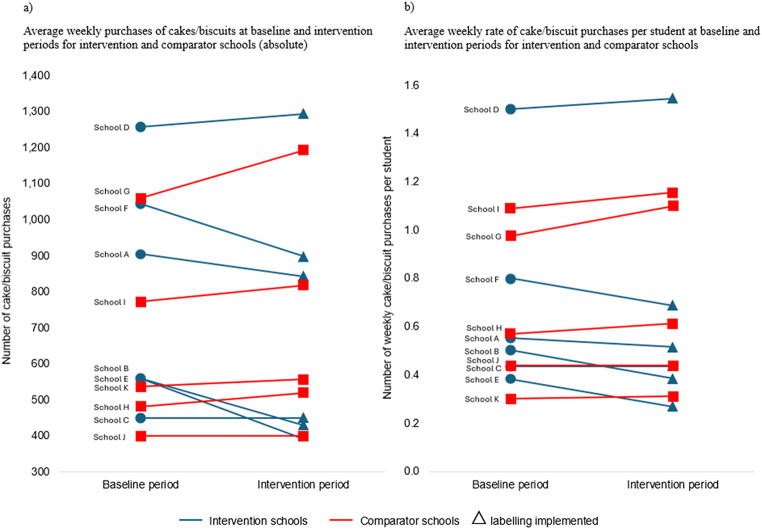
Average weekly cake/biscuit purchases (absolute) (**a**) and average weekly cake/biscuit purchase rate per student (**b**)

**Fig. 6:**
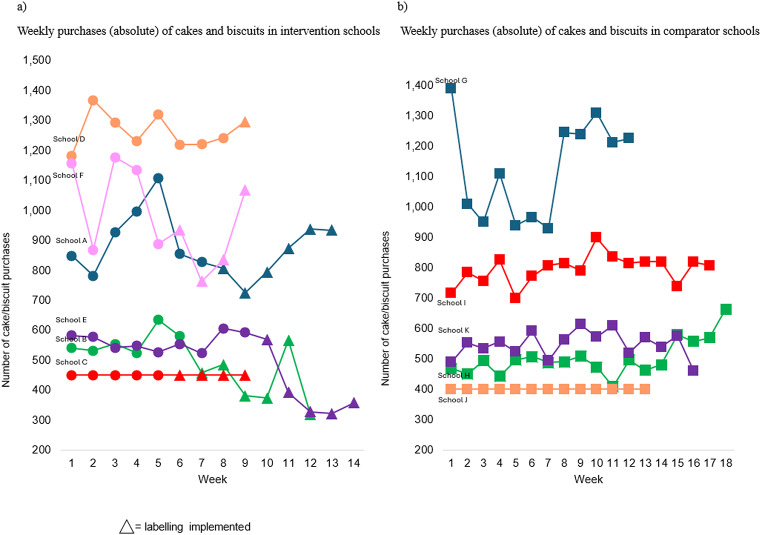
Weekly purchases (absolute) of cakes and biscuits in intervention (**a**) and comparator schools (**b**)

Figure 5 (a) shows the average weekly purchases of cakes and biscuits during the baseline and intervention period for all schools included in the analyses. This figure indicates that in four intervention schools (A, B, E and F) the average weekly purchases appeared to decrease during the intervention period compared to baseline. In the other two intervention schools, purchases either increased (D) or stayed the same (C). In contrast, in four comparator schools (G, H, I and K) purchases appeared to increase during their equivalent intervention period compared to baseline. Purchases in the remaining comparator school (J) stayed the same across baseline and intervention periods. Similar results are seen in Fig. 5 (b) which shows average weekly rate of cake/biscuit purchases per student. Figure 6 (a) and (b) reports the weekly totals of cake and biscuit purchases for intervention and comparator schools, respectively. Across timepoints, the number of purchases appear to be more stable for comparator schools compared to intervention schools. In the intervention schools where purchases appeared to decrease (A, B, E and F) there is an indication that purchases started to increase/return to baseline purchase levels over time.

#### Purchases of cakes and biscuits

Data did not violate the assumption of normality (Shapiro-Wilks test *W* (11) = 0.97, *p* = 0.85). ANCOVA showed that there was a significant between group difference in the change in number of purchases of cakes and biscuits between the two timepoints (intervention adjusted mean (standard error (se)) = 0.567 (0.02) and comparator adjusted mean (se) = 0.679 (0.02)), mean difference = -0.112, 95% CI [-0.179 to -0.045], *p* = 0.005. This is equivalent to 11.2 less cake/biscuit purchases per week per 100 students in the intervention schools compared to comparators. Sensitivity analyses did not alter the results.

### Process evaluation

#### Characteristics of PACE labelling implementation in intervention schools and fidelity assessment

The number of weeks the intervention was implemented in the six intervention schools ranged from one week to ~ six weeks. The number of cake/biscuit items the labelling was placed with ranged from two and eight cake/biscuit items that contained between 141 and 423 calories per item. All schools apart from one did not display any nutrition labelling on/near the cake/biscuit items before this study began. In this school, nutritional information was on the packaging but it was not clear to read before purchase.

Regular school visits and photographic evidence indicated the PACE labelling was in situ adequately on most occasions (on others, the calorie number was incorrect). Schools sent limited photographic evidence for most weeks in the intervention period so it is unknown if there was consistency of delivery. In all schools where contact was established, there were no reports of major issues or difficulties that impacted implementing the PACE labelling. Adaptations were made during the implementation of the PACE labelling in some schools to help the labelling displayed to be clear and visually appealing.

#### School stakeholder acceptability/school contextual factors that influenced implementation and outcomes of the PACE labelling intervention

In schools that implemented the PACE labelling in canteens, there were no major issues or difficulties that affected implementation. A major barrier to implementation were the complaints/negative feedback schools received from students, parents, senior leadership, catering manager/staff and Child and Adolescent Mental Health Services, which subsequently led to seven schools either not implementing the labelling after providing consent to participate, or making the decision to withdraw from the study after randomisation and the labelling being in place. This suggested low acceptability of the PACE labelling intervention among some school stakeholders. There appeared to be low engagement among some canteen staff in two intervention schools. This seemed to be due to them having low acceptability of the labelling because of the belief that it may have negative impacts on their students or may not have any impact on purchasing decisions. In all schools it was reported that there were no health promotion campaigns or activities for the students about nutrition and physical activity during the study that could have caused contamination of results.

## Discussion

To our knowledge, this is the first RCT to have tested the implementation of a PACE labelling intervention in secondary schools. The study assessed the efficacy of PACE labelling to reduce the purchases of cakes and biscuits by young people in secondary school canteens. This study is particularly important because most of the research that has tested the effects of PACE food labelling has taken place in laboratory settings using hypothetical food choice scenarios in adults [14]. In contrast, this study was conducted in schools, a real-world setting that is experienced by children every day. This study is also important because on average adolescents consume around two-thirds of their daily calories from ultra-processed foods, and strategies to prevent this trend are needed to protect child health [5]. Data from this study suggests that PACE labelling had a small reduction on the purchase of cakes and biscuits in the intervention schools compared to comparators. Whilst the intervention schools did not report any difficulties with implementing the PACE labelling into canteen settings, several schools withdrew from the study before or during implementation due to concerns about the negative impact this type of labelling may have on student health and well-being.

### The impact of PACE labelling on purchases of cakes and biscuits

There was a reduction in the purchases of cakes and biscuits in intervention schools compared to comparators of ~ 11 cakes/biscuits per week per 100 students. In intervention schools where purchases appeared to decrease during PACE labelling intervention period, purchases started to increase/return to baseline over time. This may show that the impact of the PACE labelling may decrease over time. The intervention was implemented for a relatively short period of six weeks and longer-term studies need to be conducted to investigate if the effects of PACE labelling are sustained.

Despite this, the findings contribute to the evidence showing PACE labelling may be an effective strategy to influence food/drink choice in the public. PACE labels may help adolescents choose healthy food and drinks [17]. PACE labelling could be easier to understand and more appealing/useful to young people compared to traffic light labelling [17], which is a common approach to food labelling in England [8]. The present study also supports previous research that found PACE labelling reduced sugary drink purchases made by adolescents in corner grocery stores [16], as well as a review showing that PACE labelling decreased the number of calories chosen from menus/consumed by the public [14].

The current study provides preliminary evidence that PACE labels may help prevent young people buying cakes and biscuits, which young people consume in excessive amounts [4] and contributes to overweight and obesity [6]. A definitive trial with a larger sample and longer follow-up is required before any firm conclusions can be made regarding the effectiveness of PACE labelling on discretionary food intake in secondary schools.

Prior to the study, none of the intervention schools displayed clear nutrition information on cakes and biscuits in their canteens. This PACE labelling study may have been a way to provide accessible/understandable energy information, in context, to help students in their food decision making. PACE labelling may have made it easier for young people to understand/interpret the energy content in the food and evaluate what consuming the item would mean for their energy balance. Eating cakes/biscuits may have become a less appealing choice to young people because these items became associated with ‘high energy cost’ in maintaining a healthy energy balance throughout the day.

Based on the findings of this study and assuming an average cake/large cookie biscuit contains 400 calories, exposure to PACE labelling could potentially reduce individuals’ calorie intake by around 45 calories per week. Over a year, that could be a reduction of 2,400 calories consumed which would equal around a third of a kilogram less weight. While the reduction in the purchasing of cakes and biscuits in the intervention schools might appear relatively small, findings relate to one food item within a single eating occasion, not a whole meal, therefore large differences in purchase data were not expected and would not be plausible. Within an overall obesity prevention strategy, PACE labelling could help to reduce purchase and consumption of discretionary foods, which in turn could prevent weight gain in young people by a small amount (or at least contribute to not increasing population weight gain). Furthermore, whilst the effects seen in this study were small, small changes in health behaviours can have important benefits to health at a population level [27]. With this in mind, if PACE labelling results in small changes to the purchasing of discretionary foods it could be a worthwhile approach to food labelling. Promoting small changes may be a more successful way to manage weight gain seen in populations compared to making large lifestyle changes, which are difficult for the public to achieve [27].

### The feasibility of implementing PACE labelling in schools

Learnings from the research have indicated that the adoption of PACE labelling in schools may be difficult, due to the low acceptability of PACE labelling among some school stakeholders (e.g. parents and school staff). Whilst this trial did not directly assess the potential for negative impacts from the PACE labelling, it is nevertheless important to note that some schools received complaints/negative feedback from school stakeholders about their potential participation in the study. A main concern raised was about the negative impacts of PACE labelling on student health and well-being, in particular, the risk of promoting or exacerbating eating disorders. This issue has been raised elsewhere with regards to PACE labelling, and calorie labelling more generally [28]. Whilst there is currently no evidence that PACE labelling leads to increases in eating disorders [29], the unintended harms of PACE labelling on health and well-being should be explored. This will certainly need to be addressed if PACE labelling were to be introduced in secondary schools or other locations.

Another issue raised by school stakeholders about PACE labelling was the view that the information on the labelling could be inaccurate and misleading because different body weights expend calories at different rates, and calories are expended at rest. Whilst both these views might be true to some degree, population averages of caloric information are used as the basis for widely used obesity prevention interventions/messages [29]. Implementing PACE labelling in schools may also be challenging because, for many school canteens, calorie information of cakes/biscuits may not be easily available as they are made on site. There may also be difficulties with displaying the labelling itself. Indeed, engagement with/acceptability of the PACE labelling among school canteen staff was mixed.

### Implications

Whilst there is a question over feasibility of the implementation of PACE food labelling in secondary schools, these findings make a unique contribution to the literature regarding the use of PACE labelling and the school environment as a context to encourage and educate young people to use nutrition information. Schools are a useful setting to deliver health behaviour change interventions as young people spend a large amount of time at school and make food selections every day in school canteens. At adolescence/secondary school age, children start to make independent decisions about what they eat, and there is a real concern about the amount of ultra-processed foods that children eat each day [5]. PACE labelling requires less hypothetical thinking, which some children and adolescents may not yet have developed [12], therefore making PACE labelling a more accessible way to help young people make decisions about what they eat. For these reasons, this age group is a key population to target in obesity prevention interventions, so that healthy habits can track into adulthood and reduce the risk of poor physical and psychological health later in life.

### Strengths and limitations of the study

This is the first study to evaluate PACE labelling implementation and efficacy in a secondary school setting in the UK. Approximately 100,000 purchase transactions were recorded in the study on which the findings are based. Another strength of the study is the inclusion of a diverse sample of schools. Schools were recruited from urban and rural areas across several regions in England with a range of deprivation status. The real-world nature of this study, and exploring the impact of PACE labelling in adolescents are further strengths. The intervention was based on evidence and theory and following six months of intervention development, to ensure the labelling used was fit for purpose. Furthermore, the development of the PACE labelling used in this trial incorporated views from young people so the labelling could be visually appealing and useful to them. A range of strategies and a substantial amount of time and resources were allocated to assess intervention fidelity.

This study has some limitations that need to be considered when interpreting the findings. Most schools recorded purchases electronically, however one school needed to record purchases manually which may have resulted in unknown recording errors. It was challenging to recruit and retain the schools involved and it was therefore not possible to limit recruitment of schools to only those that recorded transactions electronically. Though several methods were used to help identify events that may have influenced purchases of cakes/biscuits, there may have been events that were not known, although schools were asked to report all events that may have impacted school meals. One school only implemented PACE labelling for one week so these results may represent a worse case scenario and the true effects may be larger than reported here. Though a range of strategies to assess intervention fidelity were planned, it was sometimes difficult to obtain photographic evidence of the labelling in situ and keep up regular contact with the schools that were located in different towns/cities in England.

The focus of the study was to assess cake/biscuit purchases at the whole school population level therefore other energy balance behaviours (e.g. purchases of other food products, or physical activity levels) were not assessed. The change in the number of purchases of other food/drink products available to purchase was not examined. From a public health perspective these “spill-over effects” may have been positive or negative purchasing decisions. The beneficial effects of the PACE labelling in reducing purchases of cakes and biscuits (which had the study labelling in place) may have been offset by an increase in purchases of other unhealthy/energy dense items that did not have the PACE labelling (e.g. pizza, chips, other cakes/biscuits without the labelling). Conversely, displaying the PACE labelling may have resulted in a positive spill-over effect in an increase in purchases of healthier/lower calorie items (e.g. fruit).

A modest number of schools were recruited to the study which may limit the generalisability of the findings. A power or sample size calculation was not undertaken apriori. This has resulted in cautious reporting of the impact of the PACE labelling intervention to reduce purchases of cakes and biscuits here. A washout period was planned to help assess whether there was a corresponding decrease in purchases once the labelling was withdrawn, but could not be completed due to lack of data. It was not possible to assess the longer-term impacts of the PACE labelling. This would be important to establish whether the effects of PACE labelling are sustained over time and to understand both the positive and negative impacts of the labelling (e.g. on well-being). These would be important questions for future research to address. Future trials should include a longer intervention period and follow-up over a longer time frame.

## Conclusions

PACE labelling may reduce the purchases of cakes and biscuits by a small amount in young people within secondary school canteens and could be a useful strategy to help young people choose healthier food while at school. Short term implementation of PACE labelling on cakes and biscuits in schools appears generally feasible for some schools, but for others, low acceptability of PACE labelling among school stakeholders could be a barrier. Concerns raised about the potential negative effects of PACE labelling on health and well-being are important and need to be considered in future research.

## Electronic supplementary material

Below is the link to the electronic supplementary material.

Supplementary Material 1

Supplementary Material 2

## Data Availability

Data will be deposited in an appropriate data repository once the programme of research has been completed.

## Abbreviations

ANCOVA: Analysis of Covariance
IBM SPSS: IBM Statistical Package for Social Sciences
PACE: Physical activity calorie equivalent
RCT: Randomised controlled trial
UK: United Kingdom
